# Olanzapine decreased osteocyte maturation and Wnt/β-catenin signaling during loading of the alveolar bone in rats

**DOI:** 10.17305/bjbms.2022.7523

**Published:** 2023-01-06

**Authors:** Saranda Disha-Ibrahimi, Borut Furlani, Gorazd Drevenšek, Samo Hudoklin, Janja Marc, Irena Prodan Žitnik, Jakob Sajovic, Martina Drevenšek

**Affiliations:** 1 Department of Orthodontics, Faculty of Medicine, University of Ljubljana, Ljubljana, Slovenia; 2 Department of Periodontology and Oral Medicine, Faculty of Medicine, University of Prishtina, Pristina, Kosovo; 3 Institute of Pharmacology and Experimental Toxicology, Faculty of Medicine, University of Ljubljana, Ljubljana, Slovenia; 4 Institute of Cell Biology, Faculty of Medicine, University of Ljubljana, Ljubljana, Slovenia; 5 Department of Clinical Biochemistry, Faculty of Pharmacy, University of Ljubljana, Ljubljana, Slovenia; 6 Department of Orthodontics, University Medical Centre Ljubljana, Ljubljana, Slovenia

**Keywords:** Olanzapine, osteocyte maturation, bone turnover, bone modeling, Wnt/β-catenin signaling pathway, orthodontic force

## Abstract

Several studies indicate the influence of olanzapine on bone metabolism; however, the results are contradictory. We evaluated the effects of olanzapine on the Wnt/β-catenin signaling pathway, physiological alveolar bone turnover, and alveolar bone modeling due to an applied orthodontic force. Adult male rats (*n* = 48) were treated with either olanzapine or a vehicle for 21 days; then 8 rats from each group were sacrificed and the rest were divided into 4 groups: control, appliance-only, olanzapine-only, and olanzapine-appliance. The rats in the appliance groups were mounted with a superelastic closed coil spring that maintained constant orthodontic force between molars and incisors. We studied the effects of olanzapine on physiological alveolar bone turnover on day 21 of the experiment, and on alveolar bone modeling due to orthodontic force on day 56. We determined tooth movement, alveolar bone volume, activity of bone-specific cells, serum alkaline phosphatase (ALP) activity, and gene expression levels of Wnt/β-catenin signaling target genes. During forced bone modeling, olanzapine increased osteoblast volume (*P* < 0.0001) and ALP activity (*P* = 0.0011) and decreased osteoclast volume (*P* < 0.0001) and gene expression of the Wnt/β-catenin signaling target genes *Fosl1*, *Axin2*, and *Dkk1* (*P* = 0.001, *P* = 0.0076, and *P* = 0.036, respectively), and the osteocyte markers *Sost* and *Dmp1* (*P* = 0.0432 and *P* = 0.0021, respectively). Similar results were obtained during physiological alveolar bone turnover on day 21, when olanzapine downregulated the gene expression of osteocyte markers and Wnt/β-catenin signaling target genes. We concluded that olanzapine attenuated osteocyte maturation during forced bone modeling and physiological alveolar bone turnover, potentially through downregulation of the Wnt/β-catenin signaling pathway.

## Introduction

Schizophrenia and manic episodes in bipolar disorder are treated by prescribing antipsychotic drugs, among them olanzapine as an atypical antipsychotic [1, 2]. Adverse effects of the long-term use of such antipsychotic medication may lead to osteoporosis in response to hyperprolactinemia and to a decrease in bone mineral density (BMD) [3]. Due to possible effects on prolactin levels, it is generally accepted that antipsychotics are categorized as either prolactin-raising or prolactin-sparing [4–6]. Antipsychotics, besides affecting prolactin release, may influence bone metabolism through various mechanisms in the presence or absence of secondary hypogonadism, the modulation of serotonergic and adrenergic signaling, the involvement of sympathetic nervous system activity, and muscular function [7]. The effects of olanzapine on BMD have previously been studied, but the results are inconclusive [8–10]. The prolactin-sparing activity of olanzapine is favored due to the minimal loss of BMD [8, 11]. Some authors found no correlation between olanzapine and BMD [12], but found that schizophrenia itself, rather than long-term use of olanzapine, correlates with BMD loss and osteoporosis in schizophrenia patients. Some studies mention that atypical antipsychotics exhibit their pharmacological effects at least partially through the modulation of the Wnt/β-catenin signaling pathway [13], which has been shown to play a crucial role in bone remodeling by regulating osteogenic differentiation [14, 15]. Wnt/β-catenin signaling was shown to activate in osteocytes when mechanical loading was applied to the bone, and can be assumed to constitute a major signaling pathway in bone [16]. Interestingly, the Wnt pathway has been implicated in the homeostatic control of the periodontal ligament (PDL) and the alveolar bone [17], and shown to force induced bone modeling during orthodontic tooth movement (OTM) [18–20]. There is some evidence that the protein kinase b/glycogen synthase kinase-3 and Wnt/β-catenin signaling pathways may play a pivotal role in the progression of schizophrenia and in the molecular mechanisms of the action of antipsychotics [21–23] and their metabolic side effects [24]. In a study on osteoblast cultures it was shown that atypical antipsychotics, including olanzapine, could cause osteoblast apoptosis by downregulation of the Wnt/β-catenin signaling pathway [25]. However, to our knowledge, no such research has been conducted *in vivo*, nor have the effects of olanzapine on the Wnt/β-catenin signaling pathway been studied in an example of forced bone modeling. Although many studies have indicated the effects of olanzapine on bone metabolism, none have investigated its effect on bone modeling due to orthodontic force. In the present study, we evaluated the effects of olanzapine on tooth movement, alveolar bone volume, the activity of bone specific cells, and Wnt/β-catenin signaling in the alveolar bone during physiological bone turnover and bone modeling due to orthodontic force.

**Figure 1.**
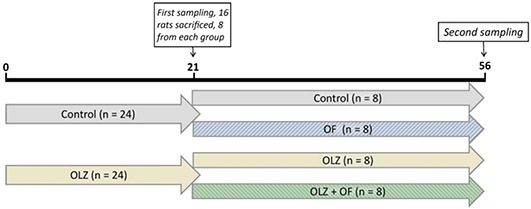
**The protocol of the study.** On the 21st day after the beginning of the experiment, the first sampling was carried out. The material needed for gene expression analysis and determining serum alkaline phosphatase activity was collected. The second sampling was carried out on the 56th day after the beginning of the experiment, the materials needed for bone histomorphometry, gene expression analysis, and serum ALP activity were obtained. OF: Appliance-only group; OLZ: Olanzapine-only group; OLZ+OF: Olanazapine-appliance group.

## Materials and methods

### Animals

Male Wistar rats (*n* = 48, 260–280 g) were used in the study, between 13 and 15 weeks of age. To avoid potential dysmetabolic changes, male rats were selected over female ones [25]. The animals were housed under standard laboratory conditions at a constant temperature (23 ^∘^C–25 ^∘^C) and humidity. A 12-h circadian cycle was established and maintained throughout the study. The rats were fed a laboratory rat chow diet (Teklad Global Rodent Diet 2016, Harlan Laboratories, An Venray, The Netherlands) and water *ad libitum*. The rat chow was available in its dry form or pre-soaked in water to enable easier food intake, as it was partially impaired due to the mounted orthodontic appliance [26, 27].

### Study protocol and olanzapine administration

At the beginning of the experiment 24 animals were treated with olanzapine 2 mg/kg daily per os for 3 weeks (Krka d.d, Novo mesto, Slovenia) and 24 animals received a vehicle for the same period of time. On the 21st day, 16 rats (OLZ21; *n* = 8 and control 21 group; *n* = 8) were sacrificed and the remaining (*n* = 32) were divided into 4 groups: control 56 group (*n* = 8), olanzapine-only group (OLZ56; *n* = 8), appliance-only group (orthodontic force – OF56; *n* = 8), and olanzapine-appliance group (OLZ+OF56; *n* = 8). The animals in both appliance groups (the OF56 and OLZ+OF56 groups) were mounted with an orthodontic appliance and continued to receive olanzapine (OLZ+OF56 group) or a vehicle (OF56 group) daily for the next five weeks (Figure 1). The entire period of olanzapine administration lasted for 8 weeks (56 days). No orthodontic appliance was placed in the animals in the control groups. All the groups were weight-matched prior to the start of the olanzapine administration. The doses were selected to reflect therapeutic concentrations and had previously shown metabolic side effects [28, 29].

### Orthodontic appliance

**Figure 2.**
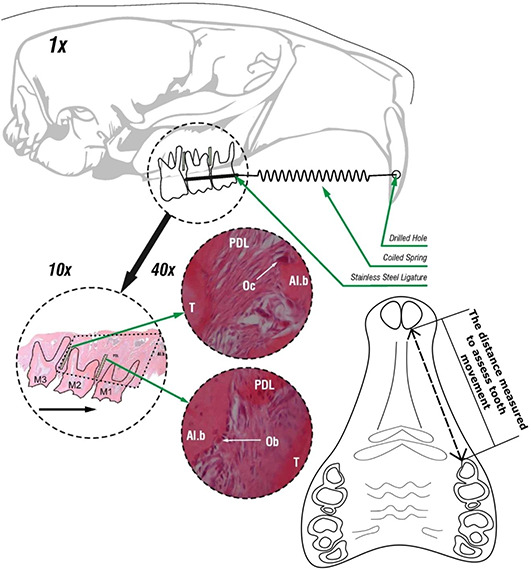
**A schematic view of the orthodontic appliance and examples of histomorphometric and tooth movement measurements carried out.Top of the figure**: an illustration of the orthodontic appliance. This consisted of a superelastic closed coil, situated between the upper first and second molar of the left side and fixed there by a stainless-steel ligature. The coil was fixed to the incisors with surgical steel wire, placed through a hole drilled in the incisors, to enhance the fixation. The hole was drilled with round, a stainless-steel bur, just above the lateral gingival margin. **Middle of the figure**: in the three dashed-line circles, tissue samples are presented under 10-fold (left) and 40-fold (right) magnification. The areas of interest in the maxilla, with 3 molars (M1–3), are shown under 10-fold magnification. The dashed rhomboid area encompassing the M2 and M1 in the 10-fold magnification circle shows the area where the alveolar bone volume was determined, and the black arrow shows the direction of tooth movement. The dot-and-dash line areas, denoted by the green arrows within the larger rhomboid surface show the regions along the mesial and distal roots of the M2, where the osteoblast and osteoclast volumes were determined. The dashed-line circles pointed to by the green arrows show a 40-fold magnification examples of osteoclasts and osteoblasts. **Bottom right of the figure**: a sketch of the upper jaw of the rat, with denoted distance that was measured to assess tooth movement. Al.b: Alveolar bone; Ob: Osteoblast; Oc: Osteoclast; PDL: Periodontal ligament; T: Tooth.

The rats from the OF56 and the OLZ-OF56 groups were equipped with an orthodontic appliance. To mount the appliance, the rats were placed under general anesthesia. Anesthesia was induced by 3 mg/kg body weight of thiopental (Tiopental, Pliva, Zagreb, Croatia), 67 mg/kg body weight of medetomidine hydrochloride (Domitor, Pfizer, Brooklyn, NY, USA), and 50 mg/kg body weight of ketamine (Bioketan, Vetoquinol Biowet, Gorzów Wielkopolski, Poland). The anesthetic mixture was injected intraperitoneally. A super-elastic closed coil spring (GAC Dentsply International, York, PA, USA), 0.15 mm in diameter and exerting a constant force of 25 cN, was placed between the first and second left maxillary molar and the incisors. A stainless-steel ligature was used to affix the spring in both groups [27]. A steel thread, to which the closed coil was attached, was placed around the first and second molars of one side of the rats’ jaw and through a hole drilled in the upper incisors of the other side. Great care was taken when drilling the hole laterally through the incisors to leave the pulp of the teeth intact, as previously described [27]. The orthodontic appliances were readjusted weekly to enable a constant amount and direction of force (Figure 2).

### Measurement of tooth movement

The measurements of tooth movement were made with a digital caliper (Wilson and Wolpert, Utrecht, The Netherlands) on the 21st and 56th days of the study, while the animals were under anesthesia [27]. The accuracy of the caliper used was ±0.01 mm. Tooth movement was measured through the distance between the mesial-most point of the left first molar of the maxilla, and the palatal-most point of the ipsilateral incisor. This measurement was done at the gingival level, in all the animal groups. Tooth movement was calculated by subtracting the distance between the teeth measured on the 56th day from the distance between the teeth measured on the 21st day. To ensure the reliability of the measurements, two independent investigators took two measurements each within a few minutes.

### Bone histomorphometry

On the 21st day after beginning of olanzapine administration, 8 rats from the OZ21 and 8 from the control 21 group were sacrificed and their right maxillary bones prepared and used for the determination of the level of gene expression. All the remaining animals (*n* = 32) were sacrificed on the 56th day, and their maxillary bones with 3 molars obtained. The left maxillary bones of the control 56 group were prepared for histomorphometric analysis. In the group of rats with the fitted orthodontic appliances, only the left maxillary bones were collected, as these had the appliances installed. Eight were collected for histomorphometric analysis.

To determine the osteoclast and osteoblast volume density, bone histomorphometry was used in all four groups. The tissue specimens were prepared by fixing them with a 4% paraformaldehyde in a phosphate-buffered saline solution with a pH between 7.2 and 7.4, kept at 14 ^∘^C for 24 h. Next, decalcification of the samples was carried out, by first submerging them in a solution of water and ethylenediamine tetraacetic acid (EDTA), at 14 ^∘^C for 12 days. The samples were then dehydrated using progressively higher solutions of ethanol, at 50%, 70%, 90%, and 100% concentration, and then with xylol. Afterward, the samples were embedded in paraffin. Finally, sections were deparaffinized and stained with hematoxylin and eosin [30].

To carry out histomorphometry, a point-counting method was used. A cycloid grid system integral to the ocular of a light microscope (BX-60, Olympus, Tokyo, Japan) was used to this end [27]. At 10-fold magnification, the area of the alveolar bone was determined, expressed as the percentage of alveolar bone area versus the tissue area consisting of tooth. PDL, connective tissue, and bone marrow spaces were determined at 10-fold magnification as well. Additionally, the osteoclast and osteoblast areas were determined, using 40-fold magnification. The areas were defined as the alveolar bone area covered with osteoblasts or osteoclasts in comparison to the total alveolar bone area, where the cells were counted in the alveolar bone alongside the mesial and distal roots of the second molar [30]. As previously described [27], the alveolar bone area, osteoblast area, and osteoclast area determined in the 20 samples of each specimen were extrapolated to assess alveolar bone volume, osteoblast volume, and osteoclast volume (Figure 2).

### Alkaline phosphatase measurement

Blood samples were obtained from the abdominal vena cava immediately before the euthanasia of the rats took place. The samples were then centrifuged and the serum isolated. The alkaline phosphatase (ALP) in the rat serum was measured using the IFCC method [31] with the commercially available kit ALP – AMP (Biosystems S.A., Spain), following the manufacturer’s instructions. Briefly, ALP catalyzes the transfer of a phosphate group from 4-nitrophenylphosphate to 2-amino-2-methyl-1-propanol (AMP), producing AMP-phosphate and 4-nitrophenol. The catalytic concentration of ALP was determined from the rate of 4-nitrophenol formation, measured at 405 nm, using a Beckman Coulter DU 730 spectrophotometer, and was referenced to a 154 mmol sodium chloride solution.

### RNA isolation and semi-quantitative RT-PCR

The maxillary bones with all three molars and their PDLs were excised and immediately frozen in liquid nitrogen. To additionally prevent the RNA from degrading, RNaseZap reagent was used during the entire procedure. The specimens were mechanically powdered using a pestle and mortar while in liquid nitrogen, to make sure the samples remained frozen throughout the procedure. After powdering, each separate specimen was transferred into a 1.5 ml tube that contained 1 ml of Trizol (TRIzol Plus RNA Purification System; Life Technologies, Carlsbad, CA, USA) to prevent the RNA from degrading. The Trizol and powdered specimen suspension was further homogenized by the use of an ultrasound homogenizer, to improve the release of RNA from the cells of the specimens. Chloroform extraction and a PureLink RNA Mini Kit (Invitrogen, Carlsbad, CA, USA) were used to isolate the RNA. Once the RNA was isolated, its quantity and quality were assessed. For the quality check, two methods were used: spectrophotometry with a NanoDrop ND-1000 Spectrophotometer (Thermo Scientific, Waltham, Mass), and capillary electrophoresis (carried out with a Bioanalyzer 2100, Agilent Technologies, Santa Clara, CA, USA). The quantity of the RNA was measured via spectrophotometry, with the NanoDrop ND-1000. The criteria for the samples to be used in our study were a suitable concentration (greater than 100 ng/µL) and an integration number in excess of 5, as determined with the Bioanalyzer 2100.

Complementary DNA was synthesized using a total of 50 ng of RNA, with the Transcriptor High Fidelity complementary DNA synthesis kit, to which the RNase was added (Life Technologies). The reaction was carried out in accordance with the manufacturers’ recommendations, with a final volume of 50 l. Predesigned assays were used for the determination of cathepsin K, osteocalcin, *Dmp1*, *Fosl1*, *Dkk1*, *Sost*, and *Axin2* gene expressions (TaqMan Gene Expression Assays, Applied Biosystems, Rn00580723_m1, Rn01455285_g1, Rn01450122_m1, Rn00564119_m1, Rn01501537_m1, Rn00577971_m1, and Rn00577441_m1, respectively). The cDNA was amplified and quantified using the Lightcycler 480 (Roche Diagnostics GmbH, Mannheim, Germany). Thermal cycling consisted of the following steps: initial cycling at 50 ^∘^C for 2 min and at 95 ^∘^C for 10 min, then 40 cycles at 95 ^∘^C for 15 s and at 60 ^∘^C for 1 min. Serial dilutions of a mix of a few samples were used to construct a standard curve. The data on cathepsin K, osteocalcin, *Dmp1*, *Fosl1*, *Dkk1*, *Sost*, and *Axin2* were normalized to the *GAPDH* expression, used as an internal housekeeping gene, to exclude variations due to different inputs of the total mRNA to the reaction. The *GAPDH* expression was determined by using the forward primer 5’TGATTCTACCCACGGCAAGTT3’, reverse primer 5’TGATGGGTTTCCCATTGATGA3’ and HOT FIREPol EvaGreen qPCR Mix Plus (no ROX) (Solis BioDyne, Tartu, Estonia), following the master mix protocol. A melting curve analysis was performed to verify the specificity of the amplification. All the reactions were carried out in duplicate, for standard samples and for the samples obtained from the four groups, with the values obtained for each reaction averaged afterwards. After normalization to the expression of *GAPDH*, a time-course-dependent consensus profile of gene expression was observed for all tested transcripts.

The osteocalcin gene expression level was used to determine osteoblast activity; the cathepsin K gene expression level as a marker of osteoclast activity; and the dentin matrix acidic phosphoprotein 1 (*Dmp1*) and sclerostin (*Sost*) gene expressions were used as osteocyte maturation markers [16]. The Wnt/β-catenin signaling pathway was evaluated by measuring the gene expression levels of the following β-catenin target genes: axis inhibition protein 2 (*Axin2*), dickkopf-related protein 1 (*Dkk1*), and fos-related antigen 1 (*Fosl1*) [32–36].

### Ethical statement

All the animal procedures and the study protocol were approved by the Ethics Committee for Animal Experiments of the Administration of the Republic of Slovenia for Food Safety, Veterinary Sector and Plant Protection (No. 34401-62/2008/20) and complied with the guiding principles in “The Care and Use of Animals.”

### Statistical analysis

Descriptive statistics [mean and standard error of the mean (SEM)] were calculated for each parameter (amount of tooth movement, alveolar bone volume, osteoblast and osteoclast volume, serum ALP activity, and gene expression levels) for all the animals in all the groups. Interexaminer reliability for tooth movement measurements was tested by the intraclass correlation coefficient (ICC), which was used to assess systematic bias. Within and between group comparisons were made for all measurements using the analysis of variance (ANOVA) and Tukey’s *post hoc* test in GraphPad Prism (GraphPad Software, San Diego, CA, USA). Comparisons between the OLZ21 and the control 21 group for all measured variables on day 21 of the experiment were performed using the unpaired t-test. Values of *P* < 0.05 were considered statistically significant. In the results, not all the groups contained the initial number of rats (*n* = 8 per group); due to a few troubled experiments some of the samples had to be excluded. A minimum of five samples per group were included in the final statistical analysis.

## Results

### Tooth movement

**Figure 3.**
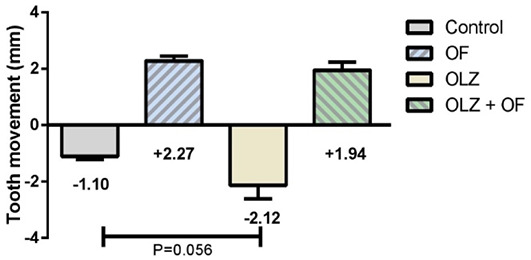
**Tooth movement on the 56th day of the experiment.** No significant differences in distal drift were found between the studied groups. The histograms depict group means, while the error bars show the group SEM. Statistical testing was carried out with the use of one-way ANOVA and Tukey’s *post hoc* tests (*n* = 5–8 animals per group). OF: Appliance-only group; OLZ: Olanzapine-only group; OLZ+OF: Olanazapine-appliance group; SEM: Standard error of the mean.

Rat molars physiologically migrate in the distal direction continuously during the animal’s lifetime—a phenomenon called distal drift, which results in an enlarged distance between molars and incisors [37]. The physiological distal drift of the molars, in the absence of OTM, was more pronounced in the OLZ56 group, but the difference was not statistically significant (*P* = 0.056). Moreover, there was no significant difference in the amount of OTM between the OF56 group and the OLZ+OF56 group (Figure 3). The overall mean value of the ICC for the measurement of the tooth movement was 0.935.

### Histomorphometric analysis

**Figure 4.**
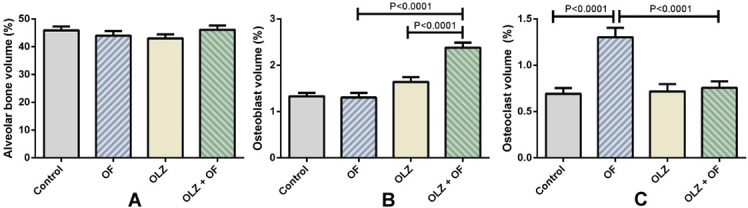
**The results of the histomorphometric analysis of the maxillary bone specimens.** (A) No significant difference between the groups was found on the 56th day of the experiment. (B) Osteoblast volume was significantly greater in the group receiving olanzapine and the orthodontic appliance (OLZ-OF56, OLZ-OF in figure) (*P* < 0.0001), when compared to the appliance-only group (OF56, OF in figure). (C) The control 56 group (control in figure, *P* < 0.0001) and the group receiving olanzapine and the orthodontic appliance (OLZ-OF, *P* < 0.0001) exhibited significantly lower osteoclast volume on the 56th day of the experiment. The data is presented as group means, with the error bars showing the SEM of the group. *n* = 5–8 animals per group. SEM: Standard error of the mean.

The histomorphometric analysis after 56 days of the experiment showed that there were no significant differences between the groups in alveolar bone volume. In the OLZ+OF56 group, a significantly higher osteoblast volume was observed than in the OLZ56 group and the OF56 group (both *P* < 0.0001). Osteoclast volume was significantly higher in the OF56 group compared to the OLZ+OF56 and the control group (both *P* < 0.0001) (Figure 4).

### ALP activity in serum

**Figure 5.**
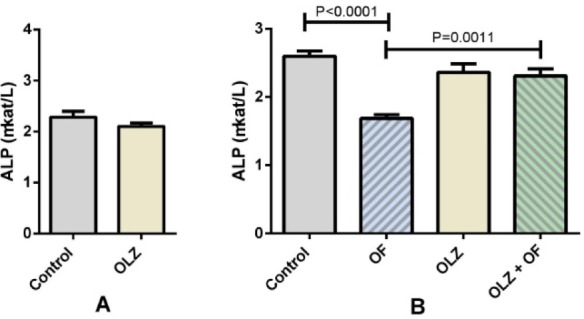
**The results of the measurements of ALP activity in the serum of the rats.** (A) No significant difference between the control 21 group (control in figure) and the olanzapine-only group (OLZ21, OLZ in figure) was observed on the 21st day of the experiment. (B) On the 56th day of the experiment, the olanzapine-appliance group 56 (OLZ+OF56, OLZ+OF in figure) exhibited significantly higher ALP activity than the appliance only group (OF56, OF in figure, *P* < 0.0011). The appliance-only group also exhibited significantly lower ALP activity levels when compared to the control group (*P* < 0.0001). The histograms depict group means, while the error bars show the group SEM. Statistical testing was carried out with the use of the unpaired t-test (A) and one-way ANOVA with Tukey’s *post hoc* tests (B) (*n* = 5–8 animals in each group). ALP: Alkaline phosphatase; SEM: Standard error of the mean.

No significant difference in the ALP activity in the rat serum was observed between the OLZ21 group and its control on the 21st day. However, on the 56th day, ALP activity was significantly higher in the OLZ+OF56 group than in the OF56 group (*P* = 0.0011), and in the control 56 group compared to the OF56 group (*P* < 0.0001) (Figure 5).

### Gene expression levels on day 21 of the experiment

**Figure 6.**
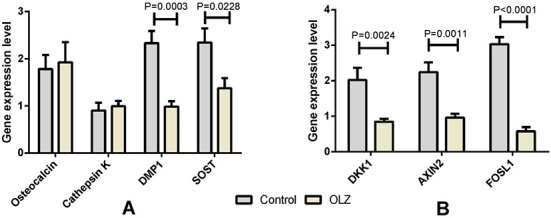
**The levels of gene expression on the 21st day of the experiment.** (A) Gene expression levels of bone cell activity markers. The osteocyte markers *Dmp1* and *Sost* were significantly downregulated in the olanzapine-only group (OLZ21, OLZ in figure) compared to the control 21 group (control in figure, *P* = 0.0003 and *P* = 0.0228, respectively). No significant differences were observed in the markers of osteoblast and osteoclast activity. (B) Gene expression levels of the Wnt/β-catenin signaling target genes. Gene expression levels of *Dkk1*, *Axin2*, and *Fosl1* were all significantly lower in the olanzapine-only group (OLZ21) compared to the control 21 group (*P* = 0.0024, *P* = 0.0011, and *P* < 0.0001). The data are presented as mean ± SEM and analyzed by the unpaired t-test (*n* = 5–8 animals in each group). DMP1: Dentin matrix acidic phosphoprotein 1; SOST: Sclerostin; DKK1: Dickkopf-related protein 1; AXIN2: Axis inhibition protein 2; FOSL1: Fos-related antigen 1; SEM: Standard error of the mean.

The results of the analysis of the gene expression levels of osteocalcin, cathepsin K, *DMP1*, and *SOST* on day 21 of the experiment are presented in Figure 6A. We observed significantly downregulated expression levels of *Dmp1* and *Sost* in the OLZ21 compared to the control 21 group (*P* = 0.0003 and *P* = 0.0228, respectively), but no significant differences were observed in the expression levels of the other two bone markers (Figure 6A). Figure 6B shows the measurements of the gene expression levels of the Wnt/β-catenin signaling target genes. The gene expression levels of *Dkk1*, *Axin2*, and *Fosl1* were all decreased in the OLZ21 group in comparison to the control 21 group (*P* = 0.0024, *P* = 0.0011, and *P* < 0.0001, respectively).

### Gene expression levels on day 56 of the experiment

**Figure 7.**
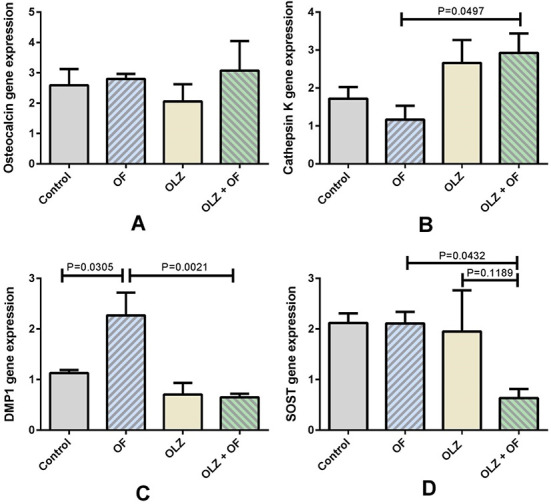
**Osteoblast, osteoclast, and osteocyte gene expression marker levels on the 56th day of the experiment.** (A) No significant differences in the osteocalcin expression level were found between the groups. (B) The expression level of cathepsin K was higher in the olanzapine appliance group (OLZ + OF56, OLZ+OF in figure) when compared to the appliance-only group (OF56, OF in figure) (*P* = 0.0497). (C) The *Dmp1* gene expression level was significantly downregulated in the olanzapine-appliance group (OLZ+OF56) compared to the appliance-only (OF56) group (*P* = 0.0021). (D) The SOST gene expression level was significantly downregulated in the olanzapine-appliance group (OLZ+OF56) compared to the appliance-only group (OF56) (*P* = 0.0432). The data are presented as mean ± SEM and analyzed by the one-way ANOVA and Tukey’s *post hoc* tests (*n* = 5–8 animals in each group). DMP1: Dentin matrix acidic phosphoprotein 1; SOST: Sclerostin; SEM: Standard error of the mean.

**Figure 8.**
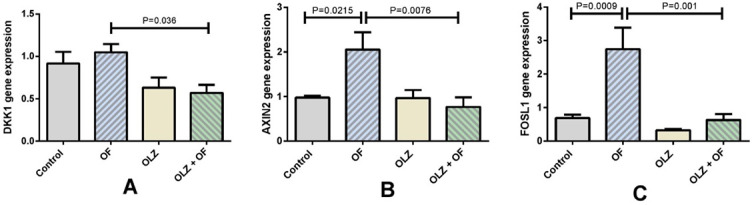
**Gene expression levels of the Wnt/β-catenin target genes on the 56th day of the experiment.** The gene expression levels of *Dkk1* (A), *Axin2* (B), and *Fosl1* (C) were all significantly downregulated in the olanzapine-appliance group (OLZ+OF56, OLZ+OF in figure) in comparison to the control group (*P* = 0.036, *P* = 0.0076, *P* = 0.001, respectively). Interestingly, the gene expression levels of *Axin2* and *Fosl1* were significantly upregulated in the appliance-only group (OF56, OF in figure) compared to the control (*P* = 0.0215 and *P* = 0.0009). This result indicates that under forced-bone modeling, the upregulation of the Wnt/β-catenin pathway represents an important biological mechanism. The data are presented as mean ± SEM and analyzed by the one-way ANOVA and Tukey’s *post hoc* tests (*n* = 5–8 animals in each group). DKK1: Dickkopf-related protein 1; AXIN2: Axis inhibition protein 2; FOSL1: fos-related antigen 1; SEM: Standard error of the mean.

The gene expression levels of osteoblast, osteoclast, and osteocyte markers are presented in Figure 7. On day 56 of the experiment, a higher gene expression level of Cathepsin K in the OLZ+OF56 group than in the OF56 group was observed (*P* = 0.0497). The gene expression levels of both osteocyte markers, *Dmp1* and *Sost*, were significantly downregulated in the OLZ+OF56 group compared to the OF56 group (*P* = 0.0021 and *P* = 0.0432, respectively). However, there were no significant differences in the gene expression levels of osteocalcin between the groups (Figure 7). On day 56 of the experiment, the gene expression levels of the Wnt/β-catenin target genes, *Axin2* and *Fosl1*, were significantly higher in the OF56 group compared to the control group, indicating the upregulation of Wnt/β-catenin signaling under the influence of the orthodontic force (*P* = 0.0215 and *P* = 0.0009, respectively). However, the gene expression levels of *Dkk1*, *Axin2*, and *Fosl1* were significantly downregulated in the OLZ+OF56 group compared to the appliance-only OF56 group (*P* = 0.036, *P* = 0.0076, and *P* = 0.001, respectively) (Figure 8). 

## Discussion

The results of the present study indicate that during forced bone modeling and physiological turnover of the alveolar bone, olanzapine downregulated the Wnt/β-catenin signaling pathway and osteocyte maturation. Moreover, under conditions of forced bone modeling olanzapine induced higher osteoblast volume and serum ALP activity, and lower osteoclast volume. But despite these histological and molecular effects, olanzapine did not cause significant changes in OTM, physiological distal drift, or alveolar bone volume. To study the role of olanzapine in forced bone modeling, we used an animal model of OTM established previously [27, 30]. OTM comprises of three stages [38], the first two lasting less than three weeks [39], and the third, late phase which represents the beginning of bone modeling and the end of the phase of necrotic tissue degradation. This process is mainly mediated by osteoblasts and osteoclasts [40]. During OTM, the process of the resorption of the alveolar bone by osteoclasts takes place. This creates space for tooth movement, while on the other side of the tooth activated osteoblasts form new bone that fills the space created by tooth movement [40]. In the late phase of OTM, which begins around 2–4 weeks after the force has been applied, tooth movement proceeds linearly through highly coordinated bone resorption and bone formation [40], and therefore presents an appropriate model to study forced bone modeling. The gene expression level for several biological molecules is quite constant in this phase of OTM, and it can be modulated by several drugs that are used clinically [27]. Selection of the time duration of the experiment must exceed four weeks to enable constant gene expression. Despite constant gene expression, changes in bone metabolism might not be significantly expressed in histological samples.

**Figure 9.**
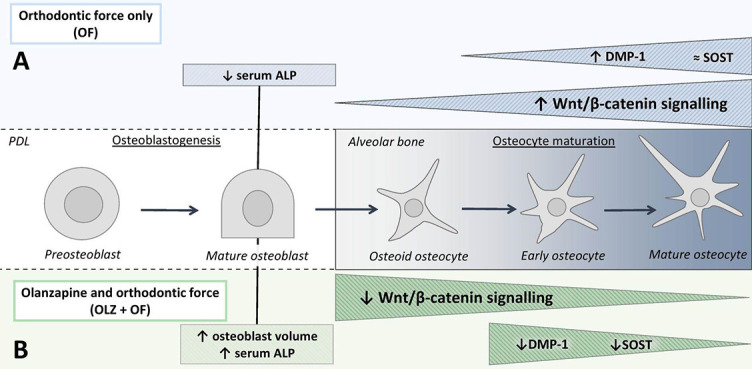
**Proposed mechanism for the effects of olanzapine on forced bone modeling.** Osteocyte maturation occurs in several stages, including osteoid osteocyte, early osteocyte, and mature osteocyte (68). During this process cells express different molecular markers. (A) Mechanical loading of the alveolar bone due to orthodontic force resulted in the upregulation of the Wnt/β-catenin signaling and gene expression of osteocyte marker *Dmp1*. (B) Olanzapine attenuated osteocyte maturation, indicated by downregulated *Dmp1* and *Sost* gene expression levels, and Wnt/β-catenin signaling during forced bone modeling in the alveolar bone. Olanzapine also increased osteoblast volume and serum ALP activity, potentially due to a slower osteoblast-to-osteocyte transition. DMP1: Dentin matrix acidic phosphoprotein 1; SOST: Sclerostin; ALP: Alkaline phosphatase.

The Wnt/β-catenin signaling pathway is a key modulator of the homeostasis of the bone [41]. Wnt/β-catenin signaling has been implicated in alveolar bone homeostasis [17] and forced bone modeling during OTM [19, 20]. In the present study, olanzapine significantly downregulated the gene expression levels of the Wnt/β-catenin signaling direct target genes, *Axin2*, *Fosl1*, and *Dkk1*, during physiological bone turnover (day 21) and during bone modeling due to orthodontic force (day 56). The inactivation of the β-catenin in mesenchymal progenitors and osteoblast precursors in the early stages of osteoblastogenesis prevents osteoblast differentiation, which results in a lack of mature osteoblasts and in the absence of bone formation [41]. But in the later stages, Wnt/β-catenin signaling inactivation in mature osteoblasts and osteocytes does not seem to affect osteoblast differentiation or bone formation, but to modulate osteoclastogenesis [16, 41, 42]. In a study by Tu et al., the activation of Wnt/β-catenin signaling in osteocytes stimulated osteoblast-to-osteocyte differentiation, upregulated the expression of osteocytic markers (*DMP1* and *SOST*), and increased the number of osteoclasts [41]. Furthermore, Wnt/β-catenin signaling has been shown to be a major pathway necessary for bone modeling, as it is activated in osteocytes when a mechanical load is applied [16, 43]. This is confirmed in the example of mice which exhibit a haploinsufficiency of β-catenin in osteocytes, where a defective anabolic response to bone loading can be observed [44]. We observed significant upregulation of *Axin2* and *Fosl1* gene expression levels in the appliance-only group compared to the control group. This indicates that the Wnt/β-catenin signaling pathway was upregulated during forced bone modeling of the alveolar bone, which represents an important physiological response to mechanical loading [19, 45]. However, we did not observe the same anabolic response to orthodontic force in groups treated with olanzapine. Olanzapine significantly decreased the gene expression levels of the Wnt/β-catenin target genes in the olanzapine-only group versus the control group (day 21) and the olanzapine-appliance group versus the appliance-only group (day 56). These observations reveal that olanzapine downregulated Wnt/β-catenin signaling in the alveolar bone during physiological alveolar bone turnover and forced bone modeling (Figure 9).

Olanzapine attenuated osteocyte maturation, evidenced by the downregulated gene expression levels of the osteocytic markers, *Dmp1* and *Sost*, during physiological bone turnover and forced modeling of the alveolar bone. *SOST* is strongly expressed by alveolar osteocytes [46, 47], and its expression is limited to mature (terminally differentiated) osteocytes [48, 49]. The gene expression of *DMP1* normally increases as osteocyte differentiation progresses to more mature osteocytes, and it is considered a marker of both early and mature osteocytes [50]. Mechanical loading has been shown to stimulate *DMP1* gene expression in the osteocytes of the alveolar bone [51], and a similar observation was made in the present study, where orthodontic force significantly upregulated the *Dmp1* gene expression level. Further, DMP1 is necessary for proper osteocyte maturation, as indicated by mice lacking DMP1, which exhibited increased osteoblastic marker expression levels, e.g., ALP and osteocalcin, and low osteocytic *Sost* expression [52]. We found increased serum ALP activity and osteoblast volume, but downregulated gene expression levels of osteocytic markers in the olanzapine-appliance group compared to the appliance-only group. Increased osteoblast volume and maintained osteoblast activity, along with significantly downregulated gene expression levels of the osteocytic markers, suggest that olanzapine attenuated osteocyte maturation, without negatively affecting osteoblast proliferation or differentiation during forced bone modeling (Figure 9). The increased osteoblast volume is related to increased osteoblast proliferation and presence of early immature osteoblasts when ALP is also normally expressed/produced [53]. However, the osteocalcin gene is mainly expressed in mature osteoblasts that are involved in the last step of bone remodeling, i.e., collagen mineralization. Our results show that olanzapine stimulated the osteoblast proliferation but had no effect on mature osteoblasts and/or on collagen mineralization.

Lower osteoclast volume in the olanzapine-appliance group compared to the appliance-only group could be a consequence of an osteocytic maturational defect. Osteocytes produce two factors that promote the proliferation and survival of osteoclasts: receptor activator of nuclear factor kappa-b ligand (RANKL) and macrophage colony-stimulating factor (M-CSF) [54, 55]. Mice lacking M-CSF in osteocytes/late osteoblasts showed impaired bone remodeling with decreased bone formation and resorption [56]. Osteocytes are thus an important source of M-CSF, which maintains osteocyte mediated bone remodeling [56]. Moreover, some studies mention that an increase in RANKL depends on SOST [41], which was substantially downregulated in the olanzapine-treated rats in our study. Therefore, it is reasonable to speculate that, due to an osteocytic maturational defect under olanzapine treatment, the osteocytes produced less RANKL and/or MCSF, ultimately resulting in a lower osteoclast volume, which was observed in the olanzapine appliance group.

The discrepancy between osteoclast volume and cathepsin K gene expression is similar to discrepancies between osteoblast volume and osteocalcin gene expression. Similar to osteocalcin in osteoblasts, cathepsin K in osteoclasts represents an effective, but not regulative protein. It is directly involved in bone tissue degradation/resorption and is expressed in mature osteoclasts. Therefore, olanzapine can exert effects on cell proliferation, expressed as decreased volume of osteoclasts, but has no effect on mature cells. Most probably olanzapine does not influence the specific gene expression of cathepsin K in mature osteoclasts. From our results, we can conclude that olanzapine possibly exerts an effect on osteoclast proliferation, but has no effect on cathepsin K gene expression in mature cells [57].

It is also possible that olanzapine influenced bone metabolism through direct modulation of its target receptors on osteoblasts and osteoclasts [58]. Olanzapine is a potent antagonist of serotonin 5HT_2A_ and 5HT_6_ receptors [59, 60], and it has been reported that pharmacological modulation of serotonin receptors affected osteoblastogenesis and osteoclastogenesis [61].

In addition, olanzapine is a partial histamine H1 receptor antagonist [62], and in our previous study, we reported that cetirizine, an H1 receptor antagonist, downregulated osteoclast volume during OTM [63]. Olanzapine could therefore partially contribute to the observed phenomena through antagonistic effects on serotonin and histamine receptors expressed on bone-specific cells. However, due to olanzapine’s numerous pharmacological targets it is difficult to pinpoint which molecular mechanism was predominant.

ALP measurement as a marker of bone formation is less specific than osteocalcin or procollagen 1 pro-peptides (P1NP). Bone mass and bone strength cannot be directly related to these biomarkers and the results cannot be interpolated to systemic changes in the skeleton. In humans, biochemical bone turnover markers should be used with caution and should always be supplemented by dual energy X-ray absorptiometry (DEXA) [64]. However, the data on both is only a surrogate marker of bone quality. The best information on bone quality is given by bone tissue structure analyses obtained by quantitative computer tomography (qCT) or histomorphometry, so animal models have an important advantage and offer trustworthy bone quality examination. In the present study, the results of bone histomorphometry are shown, and additional bone markers in the serum of animals would not substantially improve the reflection of the bone remodeling process. ALP measured in serum is a bone marker that is the most frequently used in animals, regardless of N-terminal propeptide of type I procollagen (PINP) and C-terminal telopeptide of type I collagen (CTX) being suggested by the International Federation of Clinical Chemistry and Institute of Biomedical Science in humans [64].

In the present study, osteoblast volume and serum ALP activity were increased more in the olanzapine-appliance group than in the appliance-only group, which initially does not seem to be consistent with downregulated Wnt/β-catenin signaling in the olanzapine-appliance group. However, it is probable that olanzapine attenuated Wnt/β-catenin signaling in the later stages of bone formation, during osteoblast-to-osteocyte transition and osteocyte maturation. Osteocytes are much more abundant cells than osteoblasts [50], thus having a greater contribution to the overall gene expression levels of the Wnt/β-catenin target genes. During osteoblast-to-osteocyte transition, only some osteoblasts become osteocytes [65]. It is therefore possible that due to downregulated Wnt/β-catenin signaling during osteoblast-to-osteocyte transition and osteocyte maturation, fewer osteoblast cells adopted an osteocyte fate, resulting in increased osteoblast volume and serum ALP activity. This is consistent with the significantly decreased expression of the osteocyte maturation markers, *Sost* and *Dmp1*, in the alveolar bone (Figure 9). Conclusively, in the present study, we have shown that olanzapine downregulated the Wnt/β-catenin signaling pathway, as well as interfered with osteocyte maturation during physiological bone turnover and forced bone modeling in the alveolar bone.

## Conclusion

Olanzapine downregulated the gene expression levels of the osteocyte markers, *Dmp1* and *Sost*, and the Wnt/β-catenin signaling target genes, *Fosl1*, *Axin2*, and *Dkk1*, in the alveolar bone. Treatment with olanzapine could have attenuated osteocyte maturation, potentially through downregulation of Wnt/β-catenin signaling, during both physiological alveolar bone turnover and forced bone modeling of the alveolar bone in rats.

## Ackknowledgments

We would like to thank Manja Cedilink and Nada Pavlica Dubaric for their assistance in carrying out the laboratory measurements. This work was supported by the P3-0293 research program financed by the Slovenian Research Agency and *Ad futura*—Slovenian human resources development and scholarship fund.

**Conflicts of interest:** Authors declare no conflicts of interest.

**Funding:** This work was supported by the P3-0293 research program financed by the Slovenian Research Agency and *Ad futura*—a Slovenian human resources development and scholarship fund.
